# Increased physical workload in home care service is associated with reduced recovery from work

**DOI:** 10.1007/s00420-023-01960-1

**Published:** 2023-02-18

**Authors:** Satu Mänttäri, Pihla Säynäjäkangas, Kirsikka Selander, Jaana Laitinen

**Affiliations:** 1 Finnish Institute of Occupational Health, Oulu, Finland; 2 Finnish Institute of Occupational Health, Kuopio, Finland

**Keywords:** Occupational physical demands, Elderly aid, Nurse, Heart rate variability

## Abstract

**Objective:**

This study investigated the physical workload of home care service workers and determined whether the different intensities of physical work strain experienced by home care nurses have different impacts on their recovery from work.

**Methods:**

Physical workload and recovery were measured among 95 home care nurses based on heart rate (HR) and heart rate variability (HRV) recordings during one work shift and the following night. Differences in the physical work strain were compared between younger (≤ 44-year-old) and older (≥ 45-year-old) employees and between morning and evening shifts. To determine the effects of occupational physical activity on recovery, HRV at all time points (during the workday, when awake and asleep, and whole measurement) in relation to the amount of occupational physical activity was examined.

**Results:**

The average physiological strain during the work shift, measured as metabolic equivalent (MET), was 1.8 ± 0.5. Moreover, the occupational physical demands in relation to maximal capacity were higher for the older employees. The results of the study showed that a higher occupational physical workload reduced the HRV of home care workers during the workday, leisure time, and sleep.

**Conclusions:**

These data indicate that increased occupational physical workload is associated with reduced recovery among home care workers. Therefore, decreasing occupational strain and ensuring sufficient recovery is recommended.

## Introduction

In the last thirty years, the need for elderly services has increased at the same time as market rationalities have entered the care sector (Kröger 2019). Demands for cost-effectiveness mean scarce resources and services provided by a smaller number of staff. At the same time, more frail and disabled elderly people continue to live at home. This has increased the workload as well as the physical and emotional work strain of elderly care workers (Van Aerschot et al. 2022).

Home care workers report lifting care recipients in and out of bed as the most physically strenuous feature of their work. They also report transfers into and out of chairs, toilet assistance, general housework (vacuuming, etc.), dressing, and caring for customers who require different levels of assistance as the most strenuous aspects of their work. Compared to nursing home workers, however, home care workers mostly report their work as less physically strenuous (Hasson and Arnetz 2008). Torgén et al. (1995) reported that the average physiological strain in home care work, measured as relative oxygen consumption (*VO*_2_) and heart rate (HR), did not exceed the recommended values, but that the work involved long periods of standing and walking as well as frequent awkward postures for the low back and shoulders.

The Finnish population and their caregivers are rapidly aging. Declining physical capacity and increasing age result in an increase in relative occupational physical workload (Kenny et al. 2008). Pohjonen (2001) found a significant decrease in Finnish home care workers’ perceived work ability between the ages of 40 and 44, and a further decrease after the age of 55 years. According to Merkus et al. (2019), relative muscular strain is higher among older (≥ 45-year-old) healthcare workers than among their younger counterparts, and cardiovascular load also tends to be higher. The occupational physical demands of older workers should be reduced to account for the decline in their physical capacity and to promote the work ability and work participation of older individuals (Merkus et al. 2019). At the same time, it is important to support the physical activity of workers by providing exercise interventions that are tailored according to the demands and effects of the occupation and work tasks (Mänttäri et al. 2021).

Physical and mental occupational strain impose stress on the human body. When stress is high in relation to an individual’s capacity, fatigue can occur, and recovery must be sufficient to restore work ability (Toomingas et al. 2012). Without adequate recovery, acute load effects can accumulate and lead to chronic fatigue and potentially negative health effects (Blasche et al. 2017). The need for recovery depends on the characteristics of the work performed (i.e., how physically and mentally demanding the work is) as well as on the individual and their physical and mental capacity (Toomingas et al. 2012). Wentz et al. (2020) assessed the need for recovery of engineers, carpenters, nurses, and home care nurses based on work-related fatigue symptoms after a work shift. The need for recovery score was greatest among the home care nurses, and home care nurses also formed the highest relative proportion of employees in the “high need for recovery” cluster (30 vs. 13–19% in other occupations). The home care nurses’ strategies for coping with increasing workload were thinking of work during leisure time and lowering the quality of their work to meet their work demands (Wentz et al. 2020). In a study by Blasche et al. (2017), nursing home nurses’ fatigue increased and their vigor decreased during two consecutive 12-h shifts, and at least three rest days were needed for a full recovery.

Physiological recovery from mental or physical strain can be assessed by measuring heart rate variability (HRV), which is the fluctuation in the time intervals between heartbeats. HRV is controlled by the sympathetic and parasympathetic nervous systems, and vagal outflow from the parasympathetic nervous system increases HRV (Martinmäki et al. 2006; Task Force 1996). Thus, HRV has been used as an indicator of recovery. Conversely, self-reported work stress and strain have shown to be associated with reduced HRV (Clays et al. 2011; Collins et al. 2005; Uusitalo et al. 2011; Vrijkotte et al. 2000). Reduced HRV has been related to an increased risk of cardiovascular and all-cause mortality in individuals both with and without diagnosed cardiovascular disease (Tsuji et al. 1994, 1996).

Despite the known mental and physical strain imposed by home care work (Hasson & Arnetz 2008; Muramatsu et al. 2019; Torgén et al. 1995; Van Aerschot et al. 2022), few studies have measured physiological recovery from the occupational workload. Therefore, the aim of this study was to investigate the physical workload of home care nurses and to determine whether the intensity of physical work strain has an impact on overall recovery from work measured by HRV. It is hypothesized that: (1) the objectively measured work intensity of home care nurses today is moderate (3–6 metabolic equivalents, Ainsworth et al. 2011), (2) the occupational physical demands and relative physical strain of older employees are higher than those of younger employees, and (3) there is a negative association between physical workload and the physiological recovery process, as measured by HRV parameters.

## Methods

### Participants

Ninety-five home care nurses (87 females, 8 males) volunteered to participate in this study. The women-to-men ratio approximately corresponded to the gender distribution in the health and social services in Finland, which is 7:1 (Statistics Finland 2021). Measurements were taken in nine different home care service units in Finland. Specific work shifts were selected according to the study schedule and all the employees who volunteered from these shifts were measured. Employees with a cardiac pacemaker were excluded from measurement.

The participants gave their written consent after receiving detailed information on the objectives, protocol and possible discomforts and risks. The study was approved by the Ethics Committee of the Finnish Institute for Health and Welfare (THL/1447/6.02.01/2021).

### Study design

HR and HRV of home care nurses were measured for one work shift and the following night. Seventy-five measurements were taken during the morning shift (7 am to 2 pm), 19 during the evening shift (2 pm to 9 pm), and one from an employee who worked an afternoon shift (11 am to 6 pm) (*n* = 95). The average duration of the measured work shifts was 6.8 ± 0.3 h. Two weeks later, all the employees received a *Mitä kuuluu?* questionnaire (well-being at work survey in Finnish) via email which enquired about their perceived work ability, health, and physical job demands. Sixty-six of the employees responded, giving a response rate of 69%.

### Measurements

The HR and HRV measurements were taken using Firstbeat Bodyguard 2 (Firstbeat Technologies Oy, Finland). The Firstbeat Bodyguard 2 device has been found to be valid and reliable for monitoring HRV during resting and active conditions (Palmer et al. 2021; Parak and Korhonen 2013). In addition, the Firstbeat software has been shown to estimate physical activity intensity zones (Liu et al. 2022), and *VO*_2_ with sufficient accuracy for field testing (Smolander et al. 2008, 2011).

The measurement device was attached to the participants’ chest with two single-use ECG electrodes (BlueSensor VL-00-S, Ambu, Denmark) before the start of the work shift. One electrode was attached under the clavicle on the right side and another on the rib cage on the left side. The skin was shaved of excess body hair prior to the attachment of the electrodes. The participants wore the device throughout the work shift and the rest of the day until the next morning, and were instructed to take the device off upon waking. They were told to remove the device when showering, swimming, or going to sauna. Prior to the measurement, the participants filled out a background information questionnaire (age, sex, profession, work experience, height, weight, and physical activity rating on a scale of 0–10). After the measurement, the participants reported sleep duration (time they went to bed and time they woke up) and sleep quality on a five-item scale (good–fairly good–average–fairly bad–bad).

HRV was analyzed from the duration of the workday, time spent awake and asleep, and whole measurement using the root mean square of successive differences between normal heartbeats (RMSSD). RMSSD is a widely used time-domain variable of HRV measurements. It reflects beat-to-beat variation in R-R intervals (time between consecutive heartbeats) and represents vagally-mediated changes in HRV (Shaffer and Ginsberg 2017).

HR, metabolic equivalent (MET), *VO*_2_, and energy expenditure were used as variables to represent occupational physical demands. HR, MET, *VO*_2_, and energy expenditure were calculated as averages for the work shift using the Firstbeat Lifestyle Assessment software (Firstbeat Technologies Oy, Finland) based on HR and HRV measurements (Firstbeat Technologies Oy 2012a; b). The inclusion criteria for this study data were no more than 15% erroneous R-R intervals in the recording. HR in relation to maximal heart rate (HRmax), HR in relation to HR reserve, and *VO*_2_ in relation to maximal oxygen consumption (% *VO*_2_max) were calculated on the basis of age-dependent HRmax (Eq. 1) (Jones 1988, 45) and non-exercise-calculation based *VO*_2_max values (Eq. 2) (Jackson et al. 1990).

1 $${\text{HR}}\max = 210 - 0.65 \times {\text{age}}$$

2 $$VO_{2} {\text{max}} = 56.363 + 1.921 \times {\text{activity rating}} - 0.381 \times {\text{age}} - 0.754 \times {\text{BMI}} + 10.987 \times {\text{gender}} \left( {{\text{male}} = 1, {\text{female}} = 0} \right)$$

Relative occupational physical workload was calculated as the percentage of the workday spent at 0–30% (light), 31–50% (moderate), and 51–100% *VO*_2_max (strenuous).

The *Mitä kuuluu?* questionnaire included questions about work ability, perceived health, and physical job demands. Work ability was measured using three items: the first question of the Work Ability Index (work ability compared to lifetime best on a scale of 0–10), and perceived work ability in relation to the physical and mental demands of the work (very good–good–average–fairly bad–bad) (Ahlstrom et al. 2010; Tuomi et al. 1994; van den Berg et al. 2009). Perceived health was evaluated on a five-item scale (good–fairly good–average–fairly poor–poor) (Ferrie et al. 2011; Miilunpalo et al. 1997). Last, the respondents were asked to evaluate the amount of heavy physical labor using single-item measurement (not at all or very rarely–rarely–average–often–very often) (Sabbath et al. 2012).

### Data analysis

The data were divided into two groups based on age (≤ 44 years and ≥ 45 years) and work shift (morning or evening). Age was categorized into ≤ 44 and ≥ 45 years, based on the definition of an “aging” or “older” worker (WHO 1993) and previous research on aging employees (Burr et al. 2017; Schibye et al. 2001; Soer et al. 2012). The data were also divided into three equally sized occupational physical activity groups (lowest, middle, and highest group) based on the amount of moderate to strenuous (31–100% *VO*_2_max) relative occupational physical workload. For this analysis, the amounts of relative occupational physical workload at 31–50 and 51–100% *VO*_2_max were combined because there was very little strenuous activity at 51–100% *VO*_2_max during the workdays.

Statistical analyses were carried out using SPSS Statistics 27 (IBM, USA) or R 4.4.2 (R Core Team 2022). Differences between the age groups and morning and evening shifts were compared using the Mann–Whitney *U* test. Differences in HRV (during the workday, when awake and asleep, and whole measurement RMSSD) and sleep duration between the occupational physical activity groups (lowest, middle, and highest amount of occupational physical activity) were tested using one-way analysis of variance (ANOVA) and post hoc analysis with a Bonferroni adjustment. In the ANOVA, the normal distribution of the occupational physical activity groups was tested using the Kolmogorov–Smirnov test. RMSSD during sleep did not follow a normal distribution and therefore, log transformation for the variable was performed. Correlations between HRV (during the workday, when awake and asleep, and whole measurement RMSSD) and amount of occupational physical activity (% of workday spent at 0–30 and 31–100% *VO*_2_max) were analyzed using the Pearson correlation coefficient. The level of statistical significance was set at *p* < 0.05. Results are presented as mean ± standard deviation (SD) and 95% confidence intervals (CI) for the mean between groups.

## Results

### Participant health status and work-related physical capacity

Table 1 describes the study participants. The younger employees (≤ 44) had less work experience and were in better physical condition than the older employees (≥ 45). The younger employees rated themselves as more active and healthier, although no statistically significant differences between the groups were found. The majority (53%) of all the home care nurses stated that their job included heavy physical labor often or very often, but there was no difference between the age groups’ self-rated amount of heavy physical labor.

**Table 1:** Participants’ characteristics and subjective assessment of work ability, physical activity, health status and physical job demands by all and by younger (≤ 44 years) and older (≥ 45 years) Finnish home care nurses

	Total (*n* = 95)	≤ 44 years old (*n* = 50)	≥ 45 years old (*n* = 45)	*p* value^a^	95% CI^b^
	Mean (SD)	Mean (SD)	Mean (SD)		
Age (years)	43.7 (12.0)	34.1 (7.4)	54.2 (5.3)		
Work experience (years)	10.8 (8.5)	**8.8 (6.1)**	**13.3 (10.2)**	**0.038**	**− 6.0, 0.0**
Self-rated work ability (0–10)^c^	7.6 (1.6)	7.8 (1.4)	7.4 (1.7)	0.277	0.0, 1.0
BMI^d^ (kg/m^2^)	26.8 (5.0)	27.3 (5.7)	26.3 (4.0)	0.879	**− **1.7, 2.2
Physical activity rating (0–10)	4.5 (2.0)	4.9 (2.0)	4.1 (2.0)	0.067	0.0, 2.0
VO_2_max^e^ (ml/kg/min)	29.1 (8.6)	**33.3 (8.3)**	**24.5 (6.3)**	**0.000**	**6.0, 12.1**
Self-rated health^c,f^	2.2 (0.8)	2.0 (0.7)	2.4 (0.9)	0.066	**− **1.0, 0.0
Physical job demands^c,g^	3.5 (1.1)	3.4 (1.1)	3.5 (1.0)	0.854	**− **1.0, 0.0

Significant differences are in bold

^a^*p* value of statistical significance between ≤ 44- and ≥ 45-year-old employees

^b^95% confidence interval for the mean difference between age groups

^c^*n* = 66 (total), *n* = 34 (≤ 44-year-olds), and *n* = 32 (≥ 45-year-olds)

^d^Body mass index

^e^Calculated maximal oxygen consumption

^f^Perceived health status on a scale of 1–5 (1 = good, 5 = bad)

^g^Amount of heavy physical labor on a scale of 1–5 (1 = not at all or very rarely, 5 = very often)

The average work ability rating of all the home care nurses was “good” and the work ability rating of the age groups did not differ. Work ability in relation to mental demands at work was rated as “fairly good” by both the younger and the older employees. Work ability in relation to physical work demands was also rated as “fairly good”, but the younger employees rated their physical work ability as significantly better than their older counterparts (1.9 ± 0.8 vs. 2.4 ± 0.9, respectively, *p* = 0.024, 95% CI − 1.0, 0.0).

### Occupational physical demands

Table 2 presents the occupational and physical demands of Finnish home care nurses. The overall average activity in METs (the ratio of work metabolic rate to a standard resting metabolic rate) was 1.8 ± 0.5 METs. Energy expenditure, *VO*_2_, and the average and maximal MET values were higher among the younger employees, but when compared to *VO*_2_max, the relative occupational physical workload was higher among the older employees. The older employees also spent a greater percentage of time at 51–100% of their *VO*_2_max than the younger employees. There were no differences between the age groups’ average HR, HR in relation to HRmax, or HR in relation to HR reserve.

**Table 2:** Measured physiological parameters during work shift of all and of younger (≤ 44 years) and older (≥ 45 years) Finnish home care nurses

	Total (*n* = 95)	≤ 44 years old (*n* = 50)	≥ 45 years old (*n* = 45)	*p* value^a^	95% CI^b^
	Mean (SD)	Mean (SD)	Mean (SD)		
Hear rate (HR) (bpm)	89.1 (11.7)	90.9 (11.8)	87.0 (11.4)	0.248	− 2.0, 8.0
% HRmax^c^	49.2 (6.3)	48.5 (6.1)	49.9 (6.4)	0.152	− 5.0, 1.0
% HR reserve	27.3 (7.2)	27.2 (7.3)	27.5 (7.1)	0.911	− 3.0, 3.0
MET^d^	1.8 (0.5)	**1.9 (0.6)**	**1.6 (0.4)**	**0.021**	**0.0, 0.4**
MET^d^, highest value	5.4 (1.6)	**5.9 (1.8)**	**4.8 (1.0)**	**0.004**	**0.3, 1.5**
MET^d^, highest 15 min	3.0 (0.9)	**3.2 (1.0)**	**2.7 (0.8)**	**0.027**	**0.0, 0.8**
MET^d^, highest 60 min	2.3 (0.7)	**2.5 (0.7)**	**2.1 (0.6)**	**0.008**	**0.1, 0.6**
Energy expenditure (kcal)	915.1 (255.0)	**998.0 (263.5)**	**823.0 (212.5)**	**0.001**	**41.0, 257.0**
*VO*_2_^e^ (ml/kg/min)	6.3 (1.8)	**6.8 (2.0)**	**5.7 (1.4)**	**0.012**	**0.1, 1.6**
% *VO*_2_max^f^	20.6 (4.4)	**19.5 (4.3)**	**21.7 (4.2)**	**0.038**	− **4.0, 0.0**
0–30% *VO*_2_max^f^ (% of work shift length)	86.8 (9.9)	88.5 (9.2)	84.9 (10.4)	0.078	0.0, 6.0
31–50% *VO*_2_max^f^ (% of work shift length)	11.5 (8.6)	10.5 (8.2)	12.7 (8.9)	0.210	− 5.0, 1.0
51–100% *VO*_2_max^f^ (% of work shift length)	1.4 (2.4)	**0.8 (1.9)**	**2.0 (2.8)**	**0.019**	− **1.0, 0.0**

Significant differences are in bold

^a^*p* value of statistical significance between ≤ 44- and ≥ 45-year-old employees

^b^95% confidence interval for the mean difference between age groups

^c^Percentage of maximal heart rate

^d^Metabolic equivalent

^e^Oxygen consumption

^f^Percentage of maximal oxygen consumption

Comparison of morning and evening shifts showed that the evening shift was more physically demanding. Average MET value was 1.7 ± 0.5 for the morning and 2.0 ± 0.6 for the evening shift (*p* = 0.052, 95% CI − 0.5, 0.0). Average *VO*_2_ was 6.1 ± 1.7 and 7.1 ± 2.1 ml/kg/min (*p* = 0.040, 95% CI − 1.8, 0.0) and energy expenditure 875.0 ± 248.2 and 1051.7 ± 224.8 kcal (*p* = 0.006, 95% CI − 293.0, 53.0) for the morning and evening shifts, respectively. However, there was no difference between HR or HRV during the morning and evening shifts (88.4 ± 11.6 vs. 91.8 ± 12.4 bpm, and 20.5 ± 9.7 vs. 18.1 ± 8.0 ms, respectively).

### Heart rate variability

Table 3 presents HRV (RMSSD) during the workday, when awake and asleep, and whole measurement. HRV was greatest during sleep and lowest during the workday. It was significantly lower among the older employees at all the measured time points.

**Table 3:** Heart rate variability of all and of younger (≤ 44 years) and older (≥ 45 years) Finnish home care nurses

Heart rate variability (RMSSD^a^)	Total (*n* = 95)	≤ 44 years old (*n* = 50)	≥ 45 years old (*n* = 45)	*p* value^b^	95% CI^c^
	Mean (SD)	Mean (SD)	Mean (SD)		
Workday (ms)	20.0 (9.3)	**23.0 (10.0)**	**16.6 (7.2)**	**0.001**	**3.0, 10.0**
Awake (ms)	22.3 (9.7)	**25.1 (10.3)**	**19.2 (7.9)**	**0.001**	**3.0, 10.0**
Asleep (ms)	37.0 (17.7)	**42.0 (19.1)**	**31.7 (14.6)**	**0.002**	**4.0, 17.0**
Whole measurement (ms)	27.8 (11.3)	**30.7 (11.2)**	**24.6 (10.7)**	**0.001**	**3.0, 11.0**

Significant differences are in bold

^a^Root mean square of successive R-R differences

^b^*p* value of statistical significance between ≤ 44- and ≥ 45-year-old employees

^c^95% confidence interval for the mean difference between age groups

### Associations between occupational physical workload and recovery

Workday HRV correlated with the amount of light and moderate to strenuous occupational physical activity (i.e., percentage of the workday spent at 0–30 and 31–100% *VO*_2_max) (*r* = 0.62, *p* = 0.000; *r* = − 0.62, *p* = 0.000, respectively). Similarly, HRV when awake and asleep, and whole measurement also correlated with the amount of light (*r* = 0.53, *p* = 0.000; *r* = 0.28, *p* = 0.008; *r* = 0.41, *p* = 0.000, respectively), and moderate to strenuous occupational physical activity (*r* = − 0.53, *p* = 0.000; *r* = − 0.28, *p* = 0.007; *r* = − 0.41, *p* = 0.000, respectively).

The amount of moderate to strenuous (31–100% *VO*_2_max) occupational physical activity was related to HRV during the workday, when awake, and whole measurement (Table 4). HRV during the workday and when awake differed in all three activity groups (lowest, middle, and highest amount of occupational physical activity). The whole measurement HRV of only the lowest and highest activity groups differed statistically significantly. Sleep duration or HRV while asleep of the three activity groups did not differ statistically significantly.

**Table 4:** Heart rate variability measured as RMSSD during the workday, while awake, whole measurement, and while asleep, divided into three groups (lowest, middle, and highest amount of occupational physical activity) on basis of amount of moderate to strenuous (31–100% *VO*_2_max) relative occupational physical workload

	Amount of moderate to strenuous relative occupational physical workload			95% CI^a^	95% CI^b^	95% CI^c^
	Lowest (*n* = 32)	Middle (*n* = 33)	Highest (*n* = 30)			
	Mean (SD)	Mean (SD)	Mean (SD)			
% of work shift length at 31–100% *VO*_2_max^d^	4.0 (1.9)	11.2 (2.7)	24.3 (8.9)			
RMSSD^e^, workday (ms)	**28.0 (8.0)**	18.3 (7.4)***	13.2 (5.6)***^,$^	**5.5, 14.1**	**0.7, 9.4**	**10.4, 19.2**
RMSSD^e^, awake (ms)	**28.3 (8.9)**	21.8 (9.0)**	16.5 (7.4)***^,$^	**1.3, 11.6**	**0.1, 10.5**	**6.5, 17.0**
RMSSD^e^, whole measurement (ms)	**32.3 (10.9)**	27.7 (12.1)	23.1 (8.9)**	− 1.9, 11.1	− 2.1, 11.2	**2.5, 15.8**
RMSSD^e^, asleep (ms)	41.6 (18.6)	35.7 (18.2)	33.5 (15.6)	− 4.9, 16.7	− 8.9, 13.3	− 3.1, 19.3

Significant differences are in bold

*Statistically different to “lowest” (Bonferroni post hoc adjustment), **p < 0.01, ***p < 0.001

^$^Statistically different to “middle” (Bonferroni post hoc adjustment), *p* < 0.05

^a^95% confidence interval for the mean difference between “lowest” and “middle” groups

^b^95% confidence interval for the mean difference between “middle” and “highest” groups

^c^95% confidence interval for the mean difference between “lowest” and “highest” groups

^d^Percentage of maximal oxygen consumption

^e^Root mean square of successive R-R differences (heart rate variability)

## Discussion

The results of this cross-sectional study indicate that the average estimated proportion of maximal aerobic capacity used during the workday of Finnish home care nurses is reasonably low. There was, however, an age-related difference in the balance between physical workload and capacity. A relationship was found between the level of physical workload and HRV, indicating reduced recovery from work among those working at a higher physical intensity.

Contrary to our hypothesis, the type of work in the present study sample was of reasonably low intensity, and thus, classified as light on the basis of the average MET value of 1.8 (Ainsworth et al. 2011). The average estimated *VO*_2_ did not exceed the recommended threshold limit of 33% *VO*_2_max (Bonjer 1971). This result is in accordance with a study by Torgén et al. (1995), which asserts that the average physiological strain of Swedish aides (aged 45–65) in elderly home-care services, measured as relative *VO*_2_ and HR during the workday did not exceed present recommendations. Interestingly, measured workload was in conflict with perceived strain, as 53% of employees stated that their job often involved heavy physical labor.

Increased workload and time pressure due to the increasing amount of elderly people in need of home care, financial constraints, and less staff has led to higher stress levels, and consequently, greater mental workload (Brulin et al. 2000; Denton et al. 2002; Laamanen et al. 1999). Mental workload also has an impact on the physical strain and has been shown to reduce muscular strength and endurance and impair HR recovery after exercise (Mehta and Agnew 2012). This indicates that home care workers’ increased need for recovery is not entirely due to high physical workload; it is also caused by increased time pressure and mental strain. Mental workload may also explain the discrepancy between the measured and perceived physical workload in the present study.

As hypothesized, the balance of physical workload and functional work capacity differed according to age. A decline in physical capacity is a natural mechanism of aging. If the level of occupational physical requirements remains the same, this leads to an age-related imbalance between physical capacity and demands. In this situation, older employees work at closer to their maximal capacity and their relative physical workload is higher than that of their younger counterparts (Kenny et al. 2008). According to the results of the present study, the absolute work intensity of the older home care nurses was lower than that of their younger colleagues, yet their physical workload in relation to maximal capacity was higher. The perceived amount of heavy labor was, however, similar among the older and younger employees. Unlike age, changes in physical functional capacity are not rectilinear. Changes start at different times and proceed in a different manner among different individuals (Kenny et al. 2008; Sehl and Yates 2001). Several studies of physically demanding occupations have shown that the aerobic capacity of workers declines with age, beginning at the latest after the age of 30 (Ilmarinen et al. 1991; Shvartz and Reibold 1990). Changes in musculoskeletal capacity are pronounced after the age of 45–50 (Era et al. 1992; Nygård et al. 1999). The World Health Organization has set the start of physical capacity decline at 45 years of age (WHO 1993). Typically, studies evaluating the effect of aging on physical work capacity refer to health issues, work-related illness, and injuries. The isolated influence of age-related reduction on functional work capacity has seldom been addressed, let alone that of employees in home care services. Despite lower work intensity, the higher physical workload in relation to the maximal capacity of older home care nurses is in line with previous studies and supports the recommendations to reduce physical demands as workers age (e.g., Burr et al. 2017; Pohjonen 2001). According to Mänttäri et al. (2019), overweight and obesity, together with inactivity, notably increases workload throughout the career. Therefore, the results of the present study stress the importance of a preventive approach to physical work capacity among home care workers.

Despite the differences in relative occupational physical workload, there were no statistically significant differences between the perceived overall work ability of the younger and older employees. In terms of physical work demands, however, the older home care nurses assessed their workability as weaker than their younger colleagues. On the other hand, both the younger and older home care nurses rated their work ability in relation to mental work demands similarly. Pohjonen (2001) also found an age-related decline in the perceived physical work ability of Finnish home care workers, but no differences between the psychological resources or mental work ability of the different age groups. Sustained mental work ability was assumed to be associated with extensive work experience and a wider perspective of problems that come with age (Pohjonen 2001).

HRV reflects the balance between the sympathetic and parasympathetic branches of the autonomic nervous system and is therefore, an indicator of psychophysiological stress and recovery (Kim et al. 2018). The results of the present study confirmed our hypothesis, as they showed that home care workers’ increased physical workload is associated with reduced HRV during the workday, leisure time and sleep. Consequently, the data indicate that increased physical work strain affects recovery during leisure time and sleep. This finding emphasizes the importance of shift planning (e.g., personalized work schedules) in reducing occupational strain and ensuring sufficient recovery between work shifts, especially knowing the relationship between reduced HRV and mortality (Tsuji et al. 1994, 1996). The results of this study concur with those of previous studies of blue-collar workers, in which an increased amount of occupational physical activity and elevated HR during workdays were associated with elevated resting HR and reduced nocturnal HRV, indicating impaired cardiac regulation (Hallman et al. 2017; Korshøj et al. 2021). Curiously, compared to previous studies of blue-collar workers with high levels of occupational physical activity, there was also an association between occupational physical activity and reduced HRV among home care workers with reasonably low-intensity physical strain on average. Therefore, in addition to high levels of occupational physical activity, the psychosocial nature of work (e.g., high mental demands in home care work) might also contribute to impaired autonomic cardiac modulation.

Only a few studies have examined HRV in mentally demanding home care work. HRV was lower during the work period than during total time spent awake or asleep, meaning increased sympathetic activation during working hours and conversely, increased parasympathetic activation during leisure time. This balance is important for recovery from occupational strain. The HRV of the older employees was lower during all the measured time periods. HRV is known to decline with age (Shaffer and Ginsberg 2017), but physical activity, physical fitness and body composition have also been shown to affect HRV (Teisala et al. 2014). This emphasizes the importance of physical fitness in decreasing relative occupational physical workload and promoting work ability, as well as increasing the capacity to recover from occupational strain.

This study has many strengths, such as its reasonably accurate measure of physical workload and HRV-based indicators of stress and recovery during workdays from using the ambulatory R-R interval-based method. The HRV-based method has previously been found to be valid and reliable for evaluating work-related stress and recovery (Teisala et al. 2014; van Amelsvoort et al. 2000). The use of objective measurement effectively eliminates biases in self-estimates. In addition, this study was conducted in an authentic work environment with a reasonably large sample size, providing more reliable results in a real-life situation. Nonetheless, the study also has some limitations. A longer measurement period would have increased the amount of data collected, but this was not feasible due to the irregular work schedules of the employees. In addition, to increase the proportion of measured leisure time, one day off could have been included in the measurement. HRmax and *VO*_2_max values were calculated on the basis of age, size, and self-rated physical activity, meaning that the relative occupational workload was only an estimate.

As indicated in previous studies (e.g., Torgén et al. 1995), physical strain in home care work is reasonably low in intensity. However, as the occupational physical workload is not the same for all employees, it is important to pay attention to certain risk groups. Among these risk groups are older employees, for whom relative physical strain is higher due to their decline in physical capacity, not to mention possible health problems and diseases. Home care work is typically done in shifts and these results indicate that the evening shift is more physically demanding, possibly due to less staffing. The higher physical strain of the evening shift needs to be accounted for in shift planning and the significance of recovery needs to be highlighted. Despite the difference between occupational physical strain among older and younger employees, no differences between their self-rated work ability, perceived health, or physical job demands were found. In the future, more studies with larger study samples are needed on the relationship between the objective and subjective evaluation of occupational strain.

The results of the present study confirm the need for action to prevent an early decline in work ability and, therefore, have various profound implications for employees, employers, and policymakers. Although the well-being of elderly care service personnel has long been under development, the field continues to impose a considerable burden on workers, especially because of the prevailing personnel shortage. The factors that affect physical load and recovery (age, time period of shift, intensity of work, personnel resource) should be considered when planning work shifts and dividing work tasks within work schedules. For example, working in pairs distributes the workload and, further, promotes recovery during leisure time. In addition to ergonomic shift planning, motivating personnel to promote their own health and maintain physical capacity is important, especially for older employees. Motivation and skills to maintain physical work ability throughout the elderly care service career should also be covered in education programs.

## Conclusions

The main conclusions of this study are that home care work can be classified as light-intensity work, but that the physical workload in relation to maximal capacity is higher for older Finnish home care nurses than for younger employees. This difference is mainly attributed to the age-related decline in physical capacity, as the absolute occupational physical workload was actually lower for older employees and the perceived amount of heavy labor did not differ among older and younger employees. Occupational physical strain was observed to be higher during the evening shift than during the morning shift. Higher relative occupational physical workload was associated with reduced HRV, indicating that physical workload and employee’s physical capacity affect recovery. Means to enhance recovery include ergonomic shift planning, supportive measures to maintain physical capacity, and motivation in health promotion.

## Data Availability

The datasets generated and/or analyzed during the current study are available from the corresponding author upon reasonable request.

## References

[CR1] Ahlstrom L, Grimby-Ekman A, Hagberg M, Dellve L (2010). The work ability index and single-item question: associations with sick leave, symptoms, and health – a prospective study of women on long-term sick leave. Scand J Work Environ Health.

[CR2] Ainsworth BE, Haskell WL, Herrmann SD, Meckes N, Bassett DR, Tudor-Locke C, Greer JL, Vezina J, Whitt-Glover MC, Leon AS (2011). Compendium of physical activities: a second update of codes and MET values. Med Sci Sports Exerc.

[CR3] Blasche G, Bauböck VM, Haluza D (2017). Work-related self-assessed fatigue and recovery among nurses. Int Arch Occup Environ Health.

[CR4] Bonjer FH, Parmeggiana L (1971). Energy expenditure. Encyclopedia of occupational health and safety.

[CR5] Brulin C, Winkvist A, Langendoen S (2000). Stress from working conditions among home care personnel with musculoskeletal symptoms. J Adv Nurs.

[CR6] Burr H, Pohrt A, Rugulies R, Holtermann A, Hasselhorn HM (2017). Does age modify the association between physical work demands and deterioration of self-rated general health?. Scand J Work Env Health.

[CR7] Clays E, De Bacquer D, Crasset V, Kittel F, de Smet P, Kornitzer M, Karasek R, De Backer G (2011). The perception of work stressors is related to reduced parasympathetic activity. Int Arch Occup Environ Health.

[CR8] Collins SM, Karasek RA, Costas K (2005). Job strain and autonomic indices of cardiovascular disease risk. Am J Ind Med.

[CR9] Denton M, Zeytinoglu IU, Davies S, Lian J (2002). Job stress and job dissatisfaction of home care workers in the context of health care restructuring. Int J Health Serv.

[CR10] Era P, Lyyra AL, Viitasalo J (1992). Determinants of isometric muscle strength in men of different ages. Eur J Appl Physiol Occup Physiol.

[CR11] Ferrie JE, Kivimäki M, Westerlund H, Head J, Melchior M, Singh-Manoux A, Zins M, Goldberg M, Alexanderson K, Vahtera J (2011). Differences in the association between sickness absence and long-term sub-optimal health by occupational position: a 14-year follow-up in the GAZEL cohort. Occup Environ Med.

[CR12] Firstbeat Technologies Oy (2012a) Energy expenditure estimation–firstbeat white paper. Firstbeat Technologies Oy. https://www.firstbeat.com/en/energy-expenditure-estimation-firstbeat-white-paper/ Accessed 10 Aug 2022

[CR13] Firstbeat Technologies Oy (2012b) Oxygen consumption estimation–firstbeat white paper. Firstbeat Technologies Oy. https://www.firstbeat.com/en/oxygen-consumption-estimation-firstbeat-white-paper/ Accessed 10 Aug 2022

[CR14] Hallman DM, Birk Jørgensen M, Holtermann A (2017). On the health paradox of occupational and leisure-time physical activity using objective measurements: effects on autonomic imbalance. PLoS ONE.

[CR15] Hasson H, Arnetz JE (2008). Nursing staff competence, work strain, stress and satisfaction in elderly care: a comparison of home-based care and nursing homes. J Clin Nurs.

[CR16] Ilmarinen J, Louhevaara V, Korhonen O, Nygård CH, Hakola T, Suvanto S (1991). Changes in maximal cardiorespiratory capacity among aging municipal employees. Scand J Work Environ Health.

[CR17] Jackson AS, Blair SN, Mahar MT, Wier LT, Ross RM, Stuteville JE (1990). Prediction of functional aerobic capacity without exercise testing. Med Sci Sports Exerc.

[CR18] Jones NL (1988). Clinical exercise testing.

[CR19] Kenny GP, Yardley JE, Martineau L, Jay O (2008). Physical work capacity in older adults: implications for the aging worker. Am J Ind Med.

[CR20] Kim HG, Cheon EJ, Bai DS, Lee YH, Koo BH (2018). Stress and heart rate variability: a meta-analysis and review of the literature. Psychiatry Investig.

[CR21] Korshøj M, Lund Rasmussen C, de Oliveira ST, Holtermann A, Hallman D (2021). Heart rate during work and heart rate variability during the following night: a day-by-day investigation on the physical activity paradox among blue-collar workers. Scand J Work Environ Health.

[CR22] Kröger T, Jing T-K, Kuhnle S, Pan Y, Chen S (2019). Looking for the easy way out: demographic panic and the twists and turns of long-term care policy in Finland. Aging welfare and social policy: china and the nordic countries in comparative perspective.

[CR23] Laamanen R, Broms U, Häppölä A, Brommels M (1999). Changes in the Work and motivation of staff delivering home care services in Finland. Public Health Nurs.

[CR24] Liu H, Li Q, Li Y, Wang Y, Huang Y, Bao D, Liu H, Cui Y (2022). Concurrent validity of the combined HRV/ACC sensor and physical activity diary when monitoring physical activity in university students during free-living days. Front Public Health.

[CR25] Mänttäri SK, Oksa JAH, Virkkala J, Pietilä JAK (2019). Activity level and body mass index as predictors of physical workload during working career. Saf Health Work.

[CR26] Mänttäri S, Oksa J, Lusa S, Korkiakangas E, Punakallio A, Oksanen T, Laitinen J (2021). Interventions to promote work ability by increasing physical activity among workers with physically strenuous jobs: a scoping review. Scand J Public Health.

[CR27] Martinmäki K, Rusko H, Kooistra L, Kettunen J, Saalasti S (2006). Intraindividual validation of heart rate variability indexes to measure vagal effects on hearts. Am J Physiol Heart Circ Physiol.

[CR28] Mehta RK, Agnew MJ (2012). Influence of mental workload on muscle endurance, fatigue, and recovery during intermittent static work. Eur J Appl Physiol.

[CR29] Merkus SL, Lunde LK, Koch M, Wærsted M, Knardahl S, Veiersted KB (2019). Physical capacity, occupational physical demands, and relative physical strain of older employees in construction and healthcare. Int Arch Occup Environ Health.

[CR30] Miilunpalo S, Vuori I, Oja P, Pasanen M, Urponen H (1997). Self-rated health status as a health measure: the predictive value of self-reported health status on the use of physician services and on mortality in the working-age population. J Clin Epidemiol.

[CR31] Muramatsu N, Sokas RK, Lukyanova VV, Zanoni J (2019). Perceived stress and health among home care aides: caring for older clients in a medicaid-funded home care program. J Health Care Poor Underserved.

[CR32] Nygård CH, Pohjonen T, Ilmarinen J, Ilmarinen J, Louhevaara V (1999). Muscular strength of ageing employees over an 11-year period. FinnAge—respect for the aging. People and work, research reports 26.

[CR33] Palmer AR, Distefano R, Leneman K, Berry D (2021). Reliability of the BodyGuard2 (FirstBeat) in the detection of heart rate variability. Appl Psychophysiol Biofeedback.

[CR34] Parak J, Korhonen I (2013) Accuracy of Firstbeat Bodyguard 2 beat-to-beat heart rate monitor–Firstbeat white paper. Firstbeat Technologies Oy. https://www.firstbeat.com/en/accuracy-firstbeat-bodyguard-2-heart-rate-monitor/ Accessed 12 Jan 2023

[CR35] Pohjonen T (2001). Perceived work ability of home care workers in relation to individual and work-related factors in different age groups. Occup Med (lond).

[CR36] R Core Team (2022) R: A language and environmentfor statistical computing. R Foundation forStatistical Computing, Vienna. https://www.R-project.org/

[CR37] Sabbath E, Goldberg M, Wu Q, Descath A (2012). Can a single-item measure assess physical load at work? An analysis from the GAZEL cohort. J Occup Environ Med.

[CR38] Schibye B, Hansen AF, Søgaard K, Christensen H (2001). Aerobic power and muscle strength among young and elderly workers with and without physically demanding work tasks. Appl Ergon.

[CR39] Sehl ME, Yates FE (2001). Kinetics of human aging: I. Rates of senescence between ages 30 and 70 years in healthy people. J Gerontol A Biol Sci Med Sci.

[CR40] Shaffer F, Ginsberg JP (2017). An Overview of heart rate variability metrics and norms. Front Public Health.

[CR41] Shvartz E, Reibold RC (1990). Aerobic fitness norms for males and females aged 6 to 75 years: a review. Aviat Space Environ Med.

[CR42] Smolander J, Juuti T, Kinnunen ML, Laine K, Louhevaara V, Männikkö K, Rusko H (2008). A new heart rate variability-based method for the estimation of oxygen consumption without individual laboratory calibration: application example on postal workers. Appl Ergon.

[CR43] Smolander J, Ajoviita M, Juuti T, Nummela A, Rusko H (2011). Estimating oxygen consumption from heart rate and heart rate variability without individual calibration. Clin Physiol Funct Imaging.

[CR44] Soer R, Brouwer S, Geertzen JH, van der Schans CP, Groothoff JW, Reneman MF (2012). Decline of functional capacity in healthy aging workers. Arch Phys Med Rehabil.

[CR45] Statistics Finland (Tilastokeskus) (2021). Sukupuolten tasa-arvo Suomessa 2021.

[CR46] Task Force (1996) Heart rate variability. Standards of measurement, physiological interpretation, and clinical use. Task Force of the European Society of Cardiology and the North American Society of Pacing and Electrophysiology. Circulation 93(5):1043–1065. 10.1161/01.CIR.93.5.1043 8598068

[CR47] Teisala T, Mutikainen S, Tolvanen A, Rottensteiner M, Leskinen T, Kaprio J, Kolehmainen M, Rusko H, Kujala UM (2014). Associations of physical activity, fitness, and body composition with heart rate variability-based indicators of stress and recovery on workdays: a cross-sectional study. J Occup Med Toxicol.

[CR48] Toomingas A, Mathiassen SE, Tornqvist EW, Toomingas A, Mathiassen SE, Tornqvist EW (2012). Work, working life, occupational physiology. Occupational physiology.

[CR49] Torgén M, Nygård CH, Kilbom A (1995). Physical work load, physical capacity and strain among elderly female aides in home-care service. Eur J Appl Physiol Occup Physiol.

[CR50] Tsuji H, Venditti FJ, Manders ES, Evans JC, Larson MG, Feldman CL, Levy D (1994). Reduced heart rate variability and mortality risk in an elderly cohort. The framingham heart study. Circulation.

[CR51] Tsuji H, Larson MG, Venditti FJ, Manders ES, Evans JC, Feldman CL, Levy D (1996). Impact of reduced heart rate variability on risk for cardiac events. The framingham heart study. Circulation.

[CR52] Tuomi K, Ilmarinen J, Jahkola A, Katajarinne L, Tulkki A (1994). Work ability index.

[CR53] Uusitalo A, Mets T, Martinmäki K, Mauno S, Kinnunen U, Rusko H (2011). Heart rate variability related to effort at work. Appl Ergon.

[CR54] van Aerschot L, Puthenparambil JM, Olakivi A, Kröger T (2022). Psychophysical burden and lack of support: reasons for care workers’ intentions to leave their work in the Nordic countries. Int J Soc Welf.

[CR55] van Amelsvoort LG, Schouten EG, Maan AC, Swenne CA, Kok FJ (2000). Occupational determinants of heart rate variability. Int Arch Occup Environ Health.

[CR56] van den Berg TIJ, Elders LAM, de Zwart BCH, Burdorf A (2009). The effects of work-related and individual factors on the work ability index: a systematic review. Occup Environ Med.

[CR57] Vrijkotte TG, van Doornen LJ, de Geus EJ (2000). Effects of work stress on ambulatory blood pressure, heart rate, and heart rate variability. Hypertension.

[CR58] Wentz K, Gyllensten K, Sluiter JK, Hagberg M (2020). Need for recovery in relation to effort from work and health in four occupations. Int Arch Occup Eviron Health.

[CR59] WHO (World Health Organization) (1993) Aging and working capacity. WHO, Geneva. Technical Series no. 835 8266710

